# Patient-centred interventions for drug-resistant tuberculosis: A scoping review

**DOI:** 10.4102/safp.v67i1.6180

**Published:** 2025-11-30

**Authors:** Ndiviwe Mphothulo, Marian Loveday, Hanlie Myburg

**Affiliations:** 1 Department of Nursing and Public Health, Faculty of Health Sciences, University of KwaZulu-Natal, Durban, South Africa; 2 HIV and other Infectious Diseases Research Unit (HIDRU), South African Medical Research Council, Durban, South Africa; 3 CAPRISA-MRC HIV-TB Pathogenesis and Treatment Research Unit, University of the Free State, Bloemfontein, South Africa; 4 Desmond Tutu TB Centre, Faculty of Medicine and Health Sciences, Stellenbosch University, Stellenbosch, South Africa; 5 Amsterdam Institute for Global Health and Development (AIGHD), University of Amsterdam, Amsterdam, the Netherlands

**Keywords:** social support, DR-TB, interventions, patient-centred care, patient centred interventions

## Abstract

**Background:**

People on drug-resistant tuberculosis (DR-TB) treatment face multiple challenges, which include severe disease and treatment side effects, together with psychosocial and socioeconomic challenges. These challenges impact patients’ ability to remain in care and complete their treatment.

**Methods:**

We conducted a scoping review to synthesise evidence on patient-centred care interventions that have been offered to DR-TB patients to facilitate retention in care. Studies published from 2005 until 2023 were retrieved from primary research articles, grey literature and review articles published in peer-reviewed journals.

**Results:**

Among the 347 articles sought for retrieval, 172 were subsequently excluded from the analysis for various reasons. Ultimately, 14 studies met the inclusion criteria, providing valuable insights into patient-centred interventions for DR-TB patients. These interventions aimed to mitigate the complex challenges faced by DR-TB patients during treatment and were categorised into four groups, consistent with the World Health Organization (WHO) recommendations on social support for people with DR-TB: (1) informational, (2) emotional, (3) companionship and (4) material support. Most studies (*n* = 11) offered DR-TB patients integrated forms of support. Material support was the most common form of support utilised across the studies (*n* = 12), followed by informational (*n* = 9), companionship (*n* = 7) and emotional support (*n* = 5).

**Conclusion:**

Patient-centred care interventions improve retention in care and treatment outcomes among DR-TB patients.

**Contribution:**

The study contributes to the discourse on the value of patient-centred care in managing people with DR-TB.

## Introduction

Drug-resistant tuberculosis (DR-TB) is a global health threat. In 2023, an estimated 400 000 people developed DR-TB worldwide.^1^ Globally, treatment success rates for people with DR-TB were 68% in 2021, the latest year for which treatment outcome data are available.^1^ These treatment outcomes fall short of the 75% treatment success rate target set by the World Health Organization (WHO), despite recent advances in DR-TB diagnostics and the introduction of shorter all-oral treatment regimens. Drug-resistant tuberculosis remains a threat to TB control and achievement of the End TB strategy by 2030.^2^

Drug-resistant tuberculosis occurs when *Mycobacterium tuberculosis* bacteria become resistant to the first-line drugs used to treat TB.^3^ First-line drugs to treat TB are generally safer, more affordable and have a better side effect profile compared to second-line drugs used to treat DR-TB.^3^ It was initially thought that DR-TB was because of acquired resistance in TB patients receiving inappropriate or inadequate treatment because of poor adherence, but community transmission is now considered to be responsible for the increasing burden of DR-TB.^4^ There are different types of DR-TB: Rifampicin mono-resistant TB is resistant to rifampicin but susceptible to isoniazid; multi-drug-resistant TB (MDR-TB) is resistant to both isoniazid and rifampicin. Pre-XDR TB is MDR-TB in conjunction with resistance to any fluoroquinolone (levofloxacin or moxifloxacin). Extensively drug-resistant TB (XDR-TB) is MDR-TB with resistance to the fluoroquinolones and either bedaquiline or linezolid.^5^

The human immunodeficiency virus (HIV) pandemic has fuelled drug-susceptible (DS)-TB and DR-TB, and people living with HIV (PLHIV) are at a higher risk of developing both DS- and DR-TB. Results of a systematic review and meta-analysis have shown that the risk of developing MDR-TB is 24% higher among PLHIV compared to HIV-negative individuals.^6^ The risk of mortality is increased in those with DR-TB who are co-infected with HIV.^6^

Drug-resistant tuberculosis is a serious disease requiring complex treatment with expensive and toxic medications, with a high risk of serious adverse events and sequalae.^7^ In addition, many people with DR-TB have to contend with additional barriers to care, including limited resources, poverty, overcrowding and inadequate access to health facilities,^5,6,7^ which contribute to poor retention in care. Moreover, they may experience psychosocial challenges such as stigma, discrimination and anxiety.^8,9,10,11,12,13^ These numerous challenges can contribute to suboptimal retention in care.

In recognition of these coinciding challenges, WHO introduced patient-centred care as one of the three pillars of its End TB Strategy, with the aim of facilitating high-quality care to TB patients throughout their treatment journey.^14^ Patient-centred care is defined as ‘care that is respectful of, and responsive to individual patient preferences, needs, and values’ and which ensures ‘that patient values guide all clinical decisions’.^15^ Four types of support are outlined: (1) informational support, which provides information about DR-TB disease and its treatment to patients and their families and includes training, education and counselling; (2) emotional support, which strengthens self-esteem through empathy, trust, encouragement and care; (3) companionship support, which makes an individual feel that he or she belongs to a social network that can be relied on for certain needs; and (4) material support, which includes the provision of any commodities, including financial subsidies.^16^

In this article, we review patient-centred interventions that were implemented to support DR-TB patients during treatment. We describe the type/s of patient-centred support offered and treatment outcomes, evaluate the evidence strength and consider the limitations and implications of these interventions for supporting DR-TB patients and their families.

## Research methods and design

We conducted a scoping review of published, peer-reviewed and grey literature to identify and describe patient-centred care interventions implemented globally to improve treatment outcomes for DR-TB. We followed Arksey and O’Malley’s framework for scoping reviews,^17^ to answer the research question: what patient-centred interventions have been implemented for DR-TB to improve retention in care and treatment outcomes during patients’ treatment journey? To report our scoping review process and findings, we followed the Preferred Reporting Items for Systematic Reviews and Meta-Analysis extension for scoping reviews (PRISMA-ScR).^18^ We classified the types of support that emerged from the studies according to the WHO’s definition for patient-centred care, that is material, emotional, companionship, or informational support.^16^

### Eligibility, inclusion and exclusion criteria

We included interventions published between 2005 and 2023, available in English. This period represents 10 years preceding and 8 years after the release of the WHO’s guidelines on patient-centred care for TB.^16^ This time frame allows for an assessment of interventions prior to the publication of WHO’s patient-centred guideline for TB, the impact of these guidelines on patient-centred care or social support interventions and subsequent developments in the field of patient-centred care or social support. Only intervention studies that included rifampicin-resistant TB (RR-TB), MDR-TB and/or XDR-TB, adults (≥18 years of age) and pulmonary DR-TB which reported on retention in care and/or treatment outcomes were included. We excluded the following: (1) interventions reported before 2005; (2) those with unspecified types of TB (pulmonary or extrapulmonary); (3) those with unspecified ages of participants; (4) Publications or reports not written in English; (5) non-intervention studies; and (6) those focused on paediatric and adolescent populations (< 18 years of age).

### Search strategy: Research databases and grey literature

We searched across the PubMed, EBSCOhost, Medline, Google Scholar, Web of Science, Science Direct, Africa Index, Medicus, African Journals Online (AJOL) and the WHO library databases, using multiple keyword combinations related to DR-TB, patient-centred care, or social support (see Online Appendix 1). The search strategy involved a comprehensive and iterative approach to developing keywords related to the review aim. Through repeated refinement and testing, a final set of keywords was established to capture the full spectrum of relevant results. The following keywords were used: ‘TB’ ‘OR’ ‘Tuberculosis’ OR ‘RR-TB’ OR ‘rifampicin-resistant TB’ ‘OR’ ‘MDR-TB’ OR ‘multi-drug-resistant TB’ OR ‘XDR-TB’ OR ‘extensive drug-resistant TB’ OR ‘DR-TB’ OR ‘drug-resistant TB’ OR ‘MDR-TB’ or ‘XDR-TB’ and either ‘patient-centred-care’ OR ‘socio-economic’ OR ‘psychosocial’ OR ‘economic’ OR ‘integrated care’ OR ‘nutritional support’ OR ‘psychological’ OR ‘social’ OR ‘intervention’ OR ‘patient support’ OR ‘comprehensive support’. Our grey literature searches focused on TB-related websites such as those of non-governmental agencies and global TB programmes and organisations (e.g. TB Reach, Stop TB, etc.), as well as abstract booklets from the Union World Conference on Lung Health.

### Selection of sources of evidence

All search results were exported to EndNote (version 8) and captured into Microsoft Excel. Two reviewers reviewed the records according to the specified inclusion and exclusion criteria. The process included (1) pre-screening to record the numbers of results from the multiple databases and review sources; (2) title/abstract screening to review titles and abstracts independently; (3) deletion of duplicates; (4) comparison of results to reach consensus on records to include in a full-text review; and (5) full-text review.

### Analysis and synthesis

Records were captured into Microsoft Excel using the following headings: author(s), year of publication, country where the study was conducted, intervention utilised and the impact of the intervention on the retention and/or treatment outcomes of patients on DR-TB treatment. We categorised interventions according to the types of support they offered, as outlined in the WHO’s framework introduced earlier in this article.^16^

### Ethical considerations

Ethical approval was granted by the Biomedical Research Ethics Committee (BREC), BREC/00004973/2022, of the University of KwaZulu-Natal (UKZN).

## Results

### Description of studies

**FIGURE 1:**
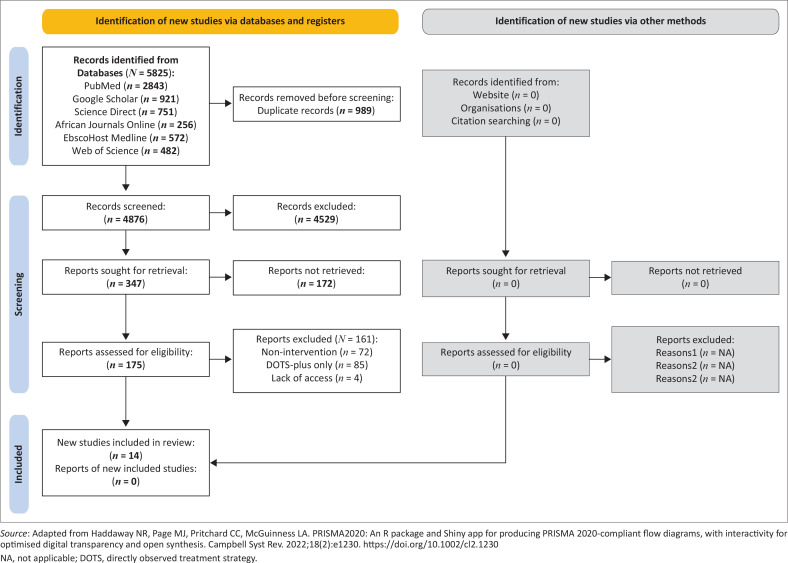
Preferred Reporting Items for Systematic Reviews and Meta-Analysis diagrams of the process and outcome of the identification and screening of literature for scoping reviews.

Our database searches yielded 4876 unique records. After an initial abstract screen, we excluded 4529 records, with 989 records removed as duplicates. Among the 347 research articles sought for retrieval, 172 of these were subsequently excluded from the analysis for various reasons (see Figure 1^19^).While 72 were non-intervention studies, 85 focused on Directly Observed Treatment Strategy Plus (DOTS-plus) and 3 records were unavailable. In all 14 records met the inclusion criteria, as illustrated in Figure 1. Our grey literature searches yielded no results, as the records did not meet the inclusion criteria.

### Characteristics of the included studies

Of the 14 research articles, 8 were based on research conducted in Asia, 3 in Africa, 2 in South America and 1 in Europe. The majority, 8/14 (57%), of the studies were published prior to the release of the WHO’s patient-centred strategy, that is between 2007 and 2014; the remaining were published after 2015. The studies used a variety of the support strategies outlined in the WHO framework.^16^ Most studies, 11 (78.%), offered integrated forms of support,^20,21,22,23,24,25,26,27,28,29,30^ with only 3 studies (21%) offering only one form of support: 2 (14%) offered material support only^31,32^ and 1 (7%) offered companionship support only.^33^ Material support was the most common form of support utilised across the studies, 12 (85%),^20,22,23,26,27,29,30,31,32^ followed by informational support (*n* = 9, 64%),^20,21,22,23,24,25,27,28,30^ companionship support (*n* = 7, 50%),^21,23,25,26,28,29,33^ and emotional support (*n* = 5, 35%).^21,22,23,24,25^

In Table 1, the country where the studies were conducted, the intervention utilised and the outcomes of the interventions are presented. Eleven studies focused on MDR-TB^20,21,23,24,25,27,29,30,32,33^ and RR-TB patients,^28^ and three studies included all types of DR-TB.^22,26,31^ Three studies included both DR-TB and DS-TB patients.^24,32,33^

**TABLE 1:** Characteristics and reported outcomes of the interventions from the included studies.

Authors	Country where study was conducted	Intervention(s) utilised	Key findings	Reported outcomes and impact of intervention
Chan et al.^27^	Taiwan	Material support - Not defined Informational support - Improved communication methods	Treatment success rate increased from 61% pre-intervention to 82% post-intervention. The intervention improved treatment success	Treatment success rate was 82% in the intervention group compared to 61% pre-intervention
Gelmanova et al.^24^	Russia	Emotional support - Expanded social support - Psychological support Material support - Nurses provided with phones to call patients - Patients assisted with procurement of social services documents	Reduced rates of patients lost to follow-up	Adherence to treatment increased by 56% in patients receiving the intervention The intervention reduced people lost to follow-up
Kunawararak et al.^33^	Thailand	Companionship support - Telephone call reminders	Improved adherence to DR-TB treatment, and improved treatment success	Adherence to treatment increased There was a 100% treatment success outcome in the intervention group
Li et al.^26^	China	Material support - Cash handouts - Nutritional supplements Companionship support - Provision of treatment supporters	Reduced LTFU rates	Intervention led to a ten-fold increase in retention in care. LTFU by 24 times
Yin et al.^30^	China	Material support - Transport fee Information support - Reminders about treatment collection - Teaching patients about coping with side effects	Improved treatment success rates	Financial support, social support and medication adherence significantly predicted a positive direct effect on treatment support Material and informational support resulted in higher treatment success rates
Oyieng’o et al.^29^	Kenya	Material support - Transport fee Companionship support - Household member supervising patient. - Home visits	Improved treatment success rates	There was 85% sputum conversion by 6 months, and 75% were cured after a follow-up of 15.5 months in the intervention group (pre- and non-intervention data not given) Material support, supervised treatment and home visits resulted in achieving the WHO target of treatment success rates
Taneja et al.^25^	India	Material support - Nutritional support (eggs and multi-nutrition) Companionship support - Home visits Informational support - Providing information to patients about DR-TB - Teaching about importance of adherence - Health education	Improved treatment success rates.	Treatment success was significantly higher in the intervention arm (p < 0.03)
Baral et al.^20^	Nepal	Material support - Monetary incentives Information support - Group counselling	Improved treatment success rate Reduced LTFU rates Reduced mortality	Treatment success rates for patients receiving counselling were 85%; for combined (counselling and financial) support, treatment success was 76%; and for those who did not receive support, it was 67% Material and informational support resulted in reduced LTFU and improved treatment success
Brust et al.^23^	South Africa	Material support - Reimbursing patients for travelling expenses Emotional support - Family workshops - Recreational excursions (Symbolic celebrations for patients’ milestones, that is, birthdays, treatment completion) Informational support - Education sessions about DR-TB Companionship - Treatment supporters	Improved treatment success rate Reduced LTFU rates	Intervention resulted in high rates of retention in care (93%) and a 77% cured/still on treatment rate Material and emotional support resulted in reduced LTFU rates
Acha et al.^21^	Peru	Material support. - Monetary incentives for transport Emotional support - Psycho-social support groups - Community mobilisation - Adherence support Companionship support - Treatment supporter - Counselling and screening for depression Informational support - Education and awareness about treatment	Improved treatment success rate Reduced LTFU	Treatment success rate was 83.5% compared to the national Peru average (65%) LTFU was 3.5% compared to the national average (17.8%)
Mohr et al.^28^	South Africa	Companionship support - Home visits Informational support - Addressing adherence barriers - Issuing pill boxes - Reviewing patient medication	Improved treatment success rates	DR-TB patients receiving support and self-administered treatment had similar outcomes to patients receiving standard of care with direct observation from healthcare workers Information and emotional support resulted in DR-TB patients retained in care without observation from healthcare workers
Sripad et al.^31^	Ecuador	Material support - Monetary incentives	Reduced LTFU rates	The LTFU rate in the study was 9.5% compared to 26.7% prior to the intervention This monetary incentive programme alleviated the economic burden of DR-TB treatment on patients
Bhatt et al.^22^	India	Material support - Cash handouts Emotional support - Motivation - Counselling Informational support - Patient–provider meetings	Improved treatment success rates. Improved treatment adherence	Treatment success was 65% in patients who received support, compared to 46% in those who did not receive support Integrated patient support increased the treatment success rate
Bhargava et al.^32^	India	Material support - Micronutrient supplements - Protein energy supplements - Food baskets	Improved treatment success rates Reduced LFTU rates	Nutritional support resulting in a 1-unit increase in BMI decreased TB mortality by 12%. A 2-unit increase in BMI resulted in a reduction in mortality by 23% 5.2% MDR-TB patients were LTFU, a retention rate of 94.7%. Compared to 10% LTFU in NTP. Food-based nutritional support was associated with improved clinical outcomes

Please see the full reference list of the article Mphothulo N, Loveday M, Myburg H. Patient-centred interventions for drug-resistant tuberculosis: A scoping review. S Afr Fam Pract. 2025;67(1), a6180. https://doi.org/10.4102/safp.v67i1.6180, for more information.

LTFU, loss to follow-up; DR-TB, drug-resistant tuberculosis; MDR-TB, multi-drug-resistant TB; TB, tuberculosis; BMI, body mass index; NTP, national TB programmes.

## Details of support given

In the following sections, we categorise the types of support included in the interventions we reviewed, as defined by the WHO. Most interventions were multifaceted and included more than one type of support.

### Theme 1: Material support

Material support was the most common type of support provided (*n* = 12).^20,21,23,24,25,26,27,28,29,30,32,33^ In Table 2, the different types of material support are listed. In interventions where monetary incentives were utilised, patients received cash to cover various essential expenses. In contrast, transport incentives provided patients with cash specifically for transportation costs or had their transport arranged directly.

**TABLE 2:** Different types of material support reported in the interventions.

Material support sub-theme	Support given
Monetary support	Cash handouts. Cash incentives for adherence. Cash to cover rent, transport and groceries.
Transport support	Cash to pay transport. Transport subsidies and reimbursement. Transport arrangement.
Nutritional support	Groceries/food support. Commodities, for example, eggs, milk. Food rations (1200 kcal and 52 g proteins per day). Micronutrient pills.

Monetary incentives were the most common form of material support utilised (*n* = 9). Cash was supplied to patients on a monthly basis for travel reimbursement,^20,22,23,26,28,29,30^ or to procure nutritional supplements^20,22,25,29^ and as a monthly incentive to reward or stimulate patients to adhere to treatment.^21,23,27,31^ A total of seven studies assisted patients with nutritional support, either as cash handouts to procure food/nutrition^20,22,25,29^ or as direct food or nutrient (micronutrient pills) supply.^22,25,26,29,32^ Bhargava et al.^32^ demonstrated that nutritional support provided to a cohort with a high prevalence of severe undernutrition, particularly in the first 2 months, resulted in decreased deaths. Other forms of material support reported included assisting patients to access social services,^27^ procuring documents to facilitate access to social services,^24^ providing cell phones to DR-TB nurses to contact patients and assisting DR-TB patients with clothing.^24^

### Theme 2: Informational support

Informational support was included in the interventions of nine studies. In Table 3, the different types of informational support are listed. Information was provided by either Community Health Workers (CHWs) or health professionals, and took place both at patients’ homes and/or health facilities. Information support included the provision of health education or disease information (*n* = 5),^21,23,24,25,27^ to individual patients or group counselling (*n* = 4),^20,21,22,28^ where patients were given an opportunity to ask about DR-TB, share their challenges and discuss how these challenges could be overcome. Five studies focused on enhancing provider–patient relationships (*n* = 5),^21,22,23,27^ three studies (*n* = 3),^16,20,22^ emphasised the importance of adherence to treatment, one study (*n* = 1) provided information on DR-TB and its treatment, and one study (*n* = 1) reminded patients to collect their medication.^30^

**TABLE 3:** Subthemes of informational support from the interventions.

Information support sub-theme	Support given
Health education	Information about DR-TB. Information about DR-TB treatment. Importance of adherence information. Teaching patients to cope with side effects. Cough etiquette health education.
Group counselling	Small group counselling sessions every 2–3 weeks. Weekly education sessions about DR-TB and HIV. Family workshops about DR-TB.
Enhancing provider–patient relationship	Regular provider–patient meetings. CHWs reporting patient challenges. Nurses encouraging patients to talk about their challenges.
Importance of adherence	Addressing barriers to adherence. Encouraging patients to report challenges. Holding sessions to talk about adherence.
Medication renewal	Reminding patients about dates to collect medication using medication renewal reminders.

DR-TB, drug-resistant tuberculosis; CHW, community health workers; HIV, human immunodeficiency virus.

### Theme 3: Emotional support

There were five interventions providing emotional support. These included counselling, psychological support,^21,22,24,25^ and motivation.^22^ Psychological support was provided in four studies (*n* = 4)^21,22,24,25^ and included addressing the emotional needs of patients, providing expanded psychological support,^21,22,24,25^ and mental and vocational rehabilitation.^15^ This support was provided by health workers (nurses, psychiatrists, psychologists and social workers) and CHWs.

### Theme 4: Companionship support

Companionship support was provided in seven studies (*n* = 7) through home visits; provision of treatment supporters; patient reminders to take medication through phone calls; and recreational activities or celebrations. Home visits were for injecting patients,^23,29^ physical assessment of patients,^29^ social situation assessments,^28^ monitoring side effects,^21^ provision of emotional support,^14,15,21^ and identifying adherence barriers.^28^ Nurses and CHWs were the cadres most often conducting home visits, with nurses being utilised for injecting patients and CHWs for social situation assessments, monitoring side effects, providing emotional support and identifying adherence barriers.

Treatment supporters usually provided companionship support (*n* = 3).^23,26,29^ Treatment supporters were either from the patient’s family or from the community. Although we have classified treatment supporters as companionship support, they also provided adherence support. Patient reminders were utilised in one study, with daily phone calls to patients to remind them to take their medication.^17^ Recreational activities or celebrations were a form of companionship provided in one study, and patients were involved in recreational activities^23^ and symbolic celebrations for patients’ birthdays, treatment completion, TB day celebrations, etc.^22^

### The outcomes of the interventions

All 14 studies reported improved treatment outcomes with patient-centred interventions, but only 10 studies had a baseline measure or control group (Table 1).

Adherence rates improved in 2 studies,^24,33^ 9 studies reported decreased LTFU rates^20,21,22,23,25,26,27,28,29,30,32,33^ and 10 studies reported an improved treatment success rate.^9,21,22,24,25,26,27,31,32,33^ We assessed the rigour and strength of the 14 studies (see Table 4) included in the review, using the Cochrane Handbook for Systematic Reviews of interventions,^34^ and found the rigour and strength of the study designs, sampling, data quality and analyses varied from moderate (*n* = 13) to strong (*n* = 1) (see Table 3).

**TABLE 4:** Table of assessment of rigour and strength of the evidence from the studies.

Study rating	Study design	Sampling	Data quality	Analysis	Global
Chan et al.^27^	Moderate	Moderate	Moderate	Moderate	Moderate
Bhatt et al.^22^	Moderate	Moderate	Moderate	Moderate	Moderate
Li et al.^26^	Moderate	Moderate	Moderate	Moderate	Moderate
Yin et al.^30^	Moderate	Moderate	Moderate	Moderate	Moderate
Oyieng’o et al.^29^	Moderate	Strong	Strong	Moderate	Moderate
Baral et al.^20^	Moderate	Moderate	Moderate	Moderate	Moderate
Brust et al.^23^	Moderate	Moderate	Moderate	Moderate	Moderate
Sripad et al.^31^	Moderate	Moderate	Moderate	Moderate	Moderate
Bhargava et al.^32^	Strong	Strong	Strong	Strong	Strong
Acha et al.^21^	Moderate	Moderate	Moderate	Moderate	Moderate
Gelmanova et al.^24^	Moderate	Moderate	Moderate	Moderate	Moderate
Taneja et al.^25^	Moderate	Moderate	Moderate	Moderate	Moderate
Kunawararak et al.^33^	Moderate	Moderate	Moderate	Moderate	Moderate
Mohr et al.^28^	Moderate	Moderate	Moderate	Moderate	Moderate

Please see the full reference list of the article Mphothulo N, Loveday M, Myburg H. Patient-centred interventions for drug-resistant tuberculosis: A scoping review. S Afr Fam Pract. 2025;67(1), a6180. https://doi.org/10.4102/safp.v67i1.6180, for more information.

## Discussion

Integrated, patient-centred care is one of three pillars in the End TB Strategy and includes the provision of patient-centred interventions.^2^ In this review, we aimed to identify and describe the patient-centred care interventions that have been implemented to support retention in care and treatment outcomes of people diagnosed with DR-TB. In this systemic review, we identified 14 studies detailing patient-centred interventions that supported people receiving DR-TB treatment and the impact of these on treatment outcomes. While 13 studies were of moderate quality,^20,21,22,23,24,25,26,27,28,29,30,31,33^ 1 study was of strong quality.^32^ However, 13 of the 14 interventions were implemented on a small scale, and none of these interventions were rolled out more broadly. Only 6/14 studies (43%) were published after the release of the WHO’s patient-centred strategy in 2015, despite the urgent need for effective patient-centred approaches to DR-TB management. This suggests a potential gap in research and implementation of patient-centred care strategies. This gap suggests a potential delay in the uptake of WHO’s recommendations in research and in practice, highlighting the need for further implementation and research efforts to accelerate the development and evaluation of patient-centred care models for people with DR-TB.

The interventions included material, informational, emotional and companionship support, and all were reported to have a positive impact on DR-TB treatment outcomes by either improving adherence or improving treatment success rates. These findings suggest that patient-centred interventions can play a role in improving DR-TB treatment outcomes. These findings align with WHO’s emphasis on addressing individual, economic, health system and social factors that influence treatment adherence and suggest that providing patient-centred care can effectively mitigate factors contributing to poor adherence, supporting WHO’s recommendation for comprehensive care for DR-TB patients.^16^

Material support was found to be a crucial component of patient-centred care, and it was the most common type of support provided (*n* = 12).^20,21,23,24,25,26,27,28,29,30,32,33^ Wen et al.^8^ had found that material support may enhance DR-TB patients’ ability to effectively leverage other forms of social support, and the provision of material support to patients from economically disadvantaged backgrounds better motivates them to adhere to treatment. However, implementation of material support has some implementation challenges, and its effects have not been fully understood by other studies. In a South African randomised control trial (RCT) providing cash vouchers for people with TB, it was found that nurses in intervention clinics failed to provide vouchers to over a third of patients because of personal biases concerning eligibility and only gave out vouchers at the end of the month, undermining the effectiveness of the intervention.^35,36^ Previous studies have explored the impact of material incentives and enablers on TB treatment outcomes. A systemic review by Lutge et al.^37^ suggests that these incentives may have short-term benefits on clinic attendance, particularly among vulnerable populations such as drug users, ex-prisoners and the homeless. However, the evidence is insufficient to determine their long-term effectiveness in improving treatment adherence.

Similarly, a review by Boccia et al.^38^ highlighted significant knowledge gaps in implementing cash transfer interventions for TB patients, underscoring a need for further research. A comprehensive review by Wells and Severn,^39^ of 11 systemic reviews and 3 overviews, found neutral to positive results for financial incentives, with no evidence of detrimental clinical effects. None of the studies included in our systematic review documented the challenges experienced with cash handouts. In our review, the importance of material support, specifically cash transfers, was underscored by several studies, which demonstrated its synergistic effect when combined with other forms of patient-centred support.^20,21,22,24,25,26,27,29^

Nutritional support emerged as an important aspect of material support, with one study demonstrating an association between nutritional support and decreased mortality.^40^ In a study by Bhargava et al.^32^ where some participants with TB had a high prevalence of undernutrition, the nutritional support delivered to patients in the form of food rations and micronutrient pills as an adjunct to anti-TB treatment resulted in significantly improved treatment outcomes and weight gain compared to the National TB Program (NTP) cohort. The mean weight gain in patients receiving nutritional support was substantial, with a weight gain of 4.8 kg, 1.5 times higher than the NTP cohort. The primarily food-based nutritional support intervention was feasible, low cost (less than $0.5 per day) and well accepted by patients.

Several studies (*n* = 7) in our review have highlighted the importance of nutritional support in improving treatment outcomes, either as cash to procure food^20,22,25,29^ or as direct food supply.^22,25,26,29,32^ The provision of nutritional support is particularly critical in resources-constrained settings, where food insecurity can exacerbate poor TB treatment outcomes.^41^ Undernutrition has been significantly associated with poor treatment outcomes, mortality and a longer period to sputum conversion among people with DR-TB,^42^ yet the existing programmatic guidance for nutritional support for people with TB is sparse.^43^

The findings of this review suggest that NTPs should consider integrating nutritional support in the DR-TB management programme.^8^ Future research should continue to explore the optimal delivery mechanisms and nutritional interventions to maximise treatment outcomes and promote patient-centred care of DR-TB patients. The importance of a multidisciplinary team approach in providing comprehensive support to DR-TB patients was also emphasised by this review. Effective patient-centred care interventions require collaboration among medical professionals, psychologists, social workers, CHWs, family members, government departments and community members.^21,22,24,25^ This coordinated effort can ensure holistic care and address the complex needs of patients with DR-TB.

The findings of this review have implications for policymakers, healthcare providers and researchers. Firstly, they emphasise the need to integrate patient-centred care support into DR-TB treatment protocols. Secondly, they highlight the importance of mobilising diverse stakeholders to provide comprehensive care. Finally, they suggest areas for future research, including the development and evaluation of innovative interventions and the examination of context-specific factors influencing intervention effectiveness.

## Limitations

This scoping review has limitations. Firstly, only 14 articles were eligible for inclusion. Secondly, there was only one RCT. More RCTs are needed to provide high-quality evidence on the effectiveness of patient-centred care support for DR-TB patients. Thirdly, some studies did not report treatment outcomes at baseline, which limited an assessment of the impact of intervention. Finally, most interventions (*n* = 13) were implemented on a small scale and in only one setting, which does little to inform implementation on a larger scale.

## Conclusion

This review provides evidence of the value of patient-centred support interventions to improve retention in care and treatment outcomes of DR-TB patients. There is evidence that material support combined with other forms of social support can improve DR-TB treatment outcomes.

The provision of nutritional support is particularly critical in improving treatment outcomes, especially in resources-constrained settings and where the patients suffer from malnutrition.

Future research should prioritise RCTs with robust evaluation and cost-effectiveness analysis together with documentation of implementation challenges experienced with more extensive implementation of these interventions, and how these were addressed, to contribute to the evidence needed to persuade NTPs to invest in such strategies. Given the impact of nutritional support on treatment outcomes recently reported, a nutritional intervention may be a starting point for NTPs.
